# Imaging algorithm and multimodality evaluation of spinal osteoblastoma

**DOI:** 10.1186/s12891-020-03252-y

**Published:** 2020-04-14

**Authors:** Zihuan Huang, Tingsong Fang, Zhiguang Si, Youcai Li, Lan Zhang, Chunling Zheng, Shenmei Li, Manting Su, Xiaomin Liu, Xiaodan Li, Yuankui Wu

**Affiliations:** 1 The First Clinical Medical College, Southern Medical University, Guangzhou, Guangdong 510515 People’s Republic of China; 2 Department of Radiology, Foshan Hospital of Traditional Chinese Medicine, Foshan, Guangdong 510515 P.R. China; 3 Department of Radiology, Dehongzhou People’s Hospital, Dehongzhou, Yunan, 678400 P.R. China; 4 Department of PET/CT Center, The First Affiliated Hospital of Guangzhou Medical University, Guangzhou, Guangdong 510000 P.R. China; 5 Department of Medical Imaging, Nanfang Hospital, Southern Medical University, No. 1838 Guangzhou Avenue North, Guangzhou, Guangzhou, Guangdong 510515 P.R. China

**Keywords:** Osteoblastoma, Spine, Radiography, CT, MRI, PET

## Abstract

**Background:**

To analyze the features of CT, MRI and PET/CT and their diagnostic value for spinal osteoblastomas (OBs).

**Methods:**

The radiological and clinical data of 21 patients with histopathologically-confirmed spinal OBs were analyzed retrospectively.

**Results:**

Sixteen of the 21 cases were benign and 5 were aggressive OBs. Tumors were located in the lumbar (*n* = 11), cervical (*n* = 4), thoracic (*n* = 5), and sacral (*n* = 1) spinal regions. Nineteen cases were centered in the posterior elements of the spine, 13 of which extended into the vertebral body. Punctate or nodular calcifications were found in all cases on CT with a complete sclerotic rim (*n* = 12) or incomplete sclerotic rim (*n* = 8). The flare phenomenon (indicative of surrounding tissue inflammation) was found in 17/21 cases on CT, thin in 11 cases and thick in 6 cases, and in 19/19 cases on MRI, thin in 1 case and thick in 18 cases. On ^18^F-FDG PET/CT, all cases (8/8) were metabolically active with the SUVmax of 12.3–16.0; the flare sign was observed in 8 cases, including 7 cases of hypometabolism and 1 case of coexistence of hypermetabolism and hypometabolism. Based on CT, 3, 12, and 6 cases were classified as Enneking stage 1, 2 and 3, respectively. Of 19 cases with MRI, 1 and 18 cases were classified as Enneking stage 2 and 3, respectively.

**Conclusions:**

Spinal OB has multiple unique characteristic radiological features. Although a larger sample size is needed, combining CT, MRI and PET may be beneficial to optimize preoperative diagnosis and care of patients with OBs.

## Background

Osteoblastoma (OB) is a rare benign bone tumor, predominately found in adolescents under the age of 20 [1]. It accounts for approximately 10% of all primary bone neoplasms, and most frequently involves the spinal column (28–36%) [1]. OB is pathologically classified into two subtypes, i.e., conventional OB and aggressive OB [2].

Diagnostic imaging plays an important role in the pre-surgical diagnosis, staging and determination of treatment options for patients with OB. Due to its low incidence, the radiologic studies on spinal OB are relatively very limited and insufficient. For instance, previous reports on spinal OB mostly used a single imaging technique with a small sample size [3–5]. Furthermore, studies with PET data are very limited [6–9]. In particular, the potential use of the flare sign (observed as the diffuse inflammatory reaction in soft tissues and marrow adjacent to bone tumors) to influence diagnosis has not been fully addressed [2, 10]. Also, the role of various imaging techniques in the management of spinal OBs is not clearly established yet [11]. Therefore, in this retrospective study, we aimed to better demonstrate the variable imaging characteristics of OBs across CT, MRI, and PET by analyzing the imaging and clinicopathological data of 21 patients with spinal OBs. These analyses might advance our understanding of how to combine CT, MRI and PET in an efficient way to increase diagnostic accuracy and aid in clinical decision-making.

## Methods

### Patients

This study was approved by the institutional review board. Using a picture archiving and communication system (PACS) developed by our hospital, from 2008 to 2019, 21 patients were identified to have a histologically-confirmed diagnosis of spinal OB. The group consisted of 15 males and 6 females, with a median age of 25.2 years and age range of 10–53 years.

### Imaging protocols

All 21 patients underwent CT scans (GE Light Speed 16 CT; Siemens Somatom Definition 64 CT) with the following parameters: 120KV, 240–320 mAs, pitch 1–1.5 mm, matrix 380*380. Nineteen patients had MRI scans (Philips Achieva 3.0 T; GE Excite HD 3.0 T), comprised of axial and sagittal T1-weighted imaging (T1WI) (TE 14–23.7 ms, TR 400–754 ms) and T2-weighted imaging (T2WI) (TE 76–138 ms, TR 3000–5100 ms), sagittal fat-suppressed T2WI (TE 80–127 ms, TR 3200–5100 ms), and axial, sagittal and coronal contrast-enhanced T1WI (TE4.6–23.4 ms, TR 189–750 ms). For all patients, contrast agent (Omniscan TM, GE Healthcare, Ireland; Magnevist, Schering, Berlin, Germany; gadopentetate dimeglumine, Consun, Guangzhou, China) was administered at a dose of 0.2 mmol/kg and a rate of 2.0–2.5 ml/s, using a power injector (Spectris Solarisl EP, Medrad, USA; TennesseeXD003, Ulrich Medical, Germany) through the antecubital vein, followed by a 20 ml sterile saline flush. Eight patients underwent 18F-FDG PET/CT examinations (Siemens, Germany), and semi-quantitative analysis was used to calculate the maximum standard uptake value (SUVmax) of tumors relative to the surrounding tissue.

### Imaging analysis

The MR examinations were evaluated visually from the hard copy images. The radiological features, including size, location, shape, density, signal intensity, contrast enhancement, sclerotic rim, flare phenomenon, uptake of 18F-FDG, and tumor staging were evaluated by three radiologists (X.X., X.X. and X.X. with 15, 9 and 4 years of experience, respectively) in concert. The assessment of the tumor staging was performed according to the Enneking staging system [12]. Discrepancies in interpretation were aligned by consensus.

## Results

The basic clinical data are summarized in Table 1. Twelve patients underwent curettage, 7 patients underwent en bloc resection, and needle biopsy alone was performed in the other 2. Pathologic examination confirmed 16 patients with conventional OB and 5 patients with aggressive OB. These tumors were located in the lumbar (*n* = 11), cervical (*n* = 4), thoracic (*n* = 5), and sacral (*n* = 1) spine. The radiologic features of CT, MRI and PET/CT of all patients are summarized in Table 2.

**Table 1:** Basic clinical data of 21 cases of spinal osteoblastomas

No./Sex	Site	CT	MRI	PET/CT	CT Staging^a^	MRI Staging	Treatment/Pathology
1/F	C5 SP	+	–	–	1	–	En B/Conventional
2/M	T11 VB + PE	+	+	–	2	2	Ne B/Conventional
3/M	T7 PE	+	+	–	1	3	En B/Conventional
4/M	C7 VB	+	+	–	3	3	Curettage/Conventional
5/M	L5 VB + PE	+	–	+	2	–	Curettage/Conventional
6/M	T4 VB + PE	+	+	–	2	3	Curettage/Conventional
7/F	T3 VB + PE	+	+	–	2	3	Curettage/Conventional
8/M	C2 VB + PE	+	+	+	2	3	Curettage/Conventional
9/M	L4 VB + PE	+	+	–	3	3	Curettage/Conventional
10/M	L4 VB + PE	+	+	–	2	3	Curettage/Conventional
11/M	T5 PE	+	+	–	3	3	En B/Conventional
12/F	T8 PE	+	+	–	2	3	En B/Conventional
13/M	L1 VB + PE	+	+	–	3	3	Curettage/Conventional
14/M	C1–2 VB + PE	+	+	+	3	3	Curettage/Aggressive
15/F	T3 VB + PE	+	+	+	2	3	Curettage/Aggressive
16/F	T2 VB + PE	+	+	+	3	3	Ne B/Aggressive
17/M	L4 PE	+	+	+	2	3	En B/Conventional
18/M	L2 PE	+	+	+	2	3	En B/Conventional
19/F	S2 PE	+	+	+	2	3	En B/Conventional
20/M	L4 VB + PE	+	+	–	1	3	Curettage/Aggressive
21/M	T4 VB + PE	+	+	–	2	3	Curettage/Aggressive

*SP* spinous process, *VB* vertebral body, *PE* posterior elements, *+* available, *−* not available, ^a^ Enneking staging with CT, En B en bloc resection, Ne B needle biopsy

**Table 2:** Imaging features of 21 cases of spinal osteoblastomas

No.	Size^a^ (mm)	Shape	Calcification	Sclerotic rim	Flare tissues on CT	Flare tissues on MRI	SUVmax^b^
1	22	Oval	Punctate	Complete	Absent	–	–
2	29	Oval	Punctate	Complete	Thin, clear	Thin, clear	–
3	24	Irregular	Punctate	Complete	Absent	Thick, blur	–
4	35	Irregular	Punctate	Complete	Thick, blur	Thick, blur	–
5	38	Irregular	Punctate	Incomplete	Thin, clear	–	14.3
6	40	Irregular	Punctate	Complete	Thin, blur	Thick, blur	–
7	27	Irregular	Nodular	Complete	Thin, blur	Thick, blur	–
8	39	Irregular	Punctate	Complete	Thin, blur	Thick, blur	14.8
9	63	Lobulated	Nodular	Incomplete	Thick, blur	Thick, blur	–
10	46	Lobulated	Nodular	Complete	Thin, blur	Thick, blur	–
11	58	Lobulated	Nodular	Incomplete	Thick, blur	Thick, blur	–
12	38	Lobulated	Nodular	Complete	Thin, blur	Thick, blur	–
13	32	Irregular	Nodular	Incomplete	Thick, blur	Thick, blur	–
14	27	Irregular	Punctate	Incomplete	Thick, blur	Thick, blur	15.3
15	45	Lobulated	Nodular	Complete	Thin, blur	Thick, blur	16.0
16	68	Lobulated	Nodular	Incomplete	Thick, blur	Thick, blur	15.7
17	28	Lobulated	Nodular	Incomplete	Thin, blur	Thick, blur	12.3
18	23	Irregular	Punctate	Absent	Absent	Thick, blur	13.6
19	50	Oval	Punctate	Incomplete	Thin, blur	Thick, blur	15.1
20	53	Irregular	Nodular	Complete	Absent	Thick, blur	–
21	48	Irregular	Punctate	Complete	Thin, blur	Thick, blur	–

^a^ Maximum diameter of lesion on transverse section, ^b^ SUV of bone lesions, − not available

### General radiological features

**Fig. 1:**
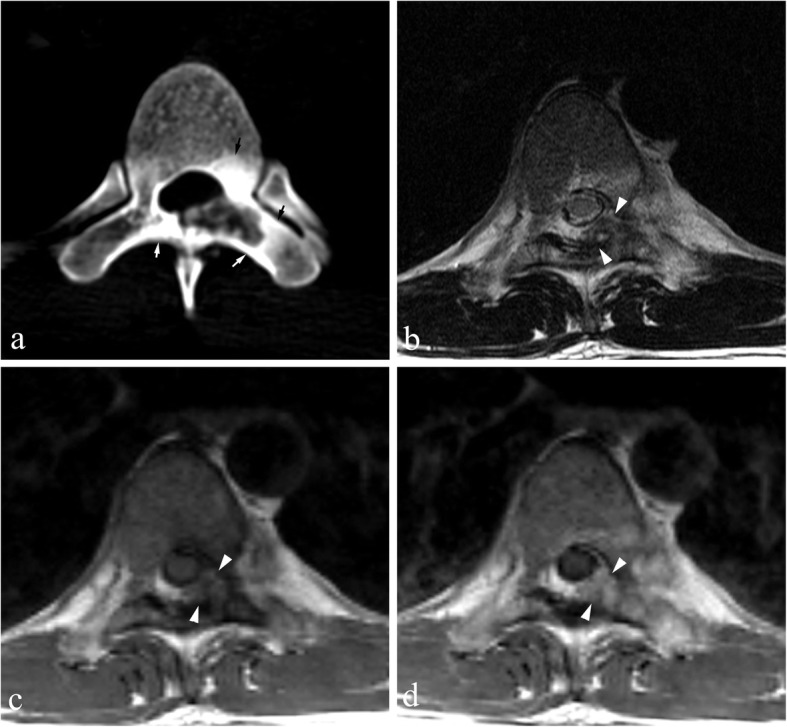
Osteoblastoma in a 25-year-old male, Enneking stage 3 on both CT and MRI. **a** and **b** Axial CT soft tissue and bone windows show a lesion causing extensive osteolytic destruction in L4 (which mainly affects the left pedicle, traverse process and lamina) with an incomplete sclerotic rim, unclear borders, and spotted calcifications. Note the thin flares in the epidural space (arrow). **c** and **d** Axial MRI T2WI and contrast-enhanced T1WI demonstrate the flare phenomenon adjacent to the tumor, i.e., abnormal swollen soft tissues in the spinal canal compressing the dural sac (arrow) lateral to the spinous process (arrowheads), showing hyperintensity on T2WI and marked enhancement with contrast

**Fig. 2:**
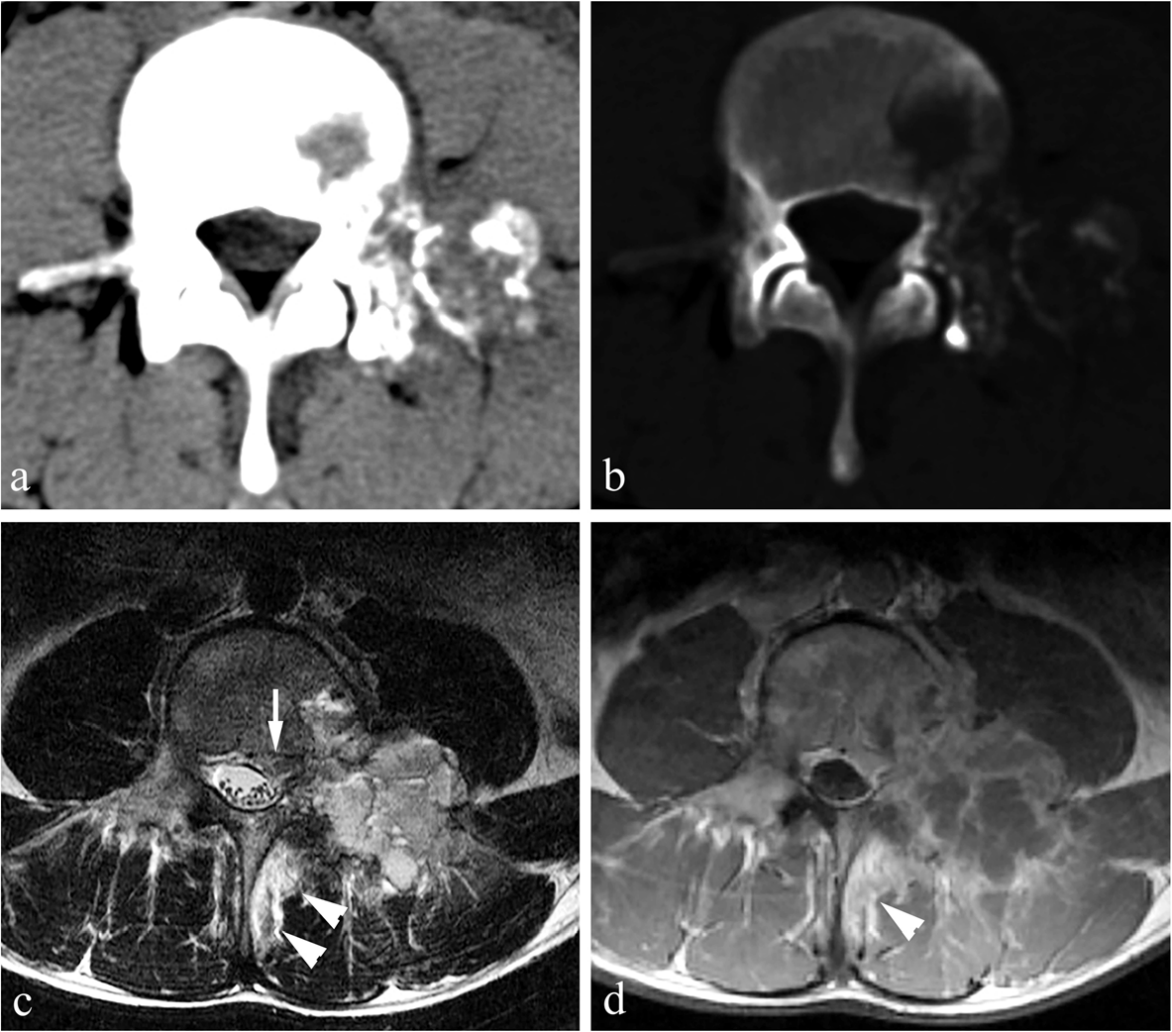
Osteoblastoma in a 29-year-old male, Enneking stage 2 on CT and stage 3 on MRI. **a** Axial CT bone window shows expansile bone destruction in the left lamina of L7 with a clear sclerotic rim and speckled calcifications. Note sclerotic changes (arrows) in the surrounding areas. Axial MRI T2WI (**b**), T1WI (**c**), and contrast-enhanced T1WI (**d**) show a soft tissue mass in the epidural space (arrowheads)

**Fig. 3:**
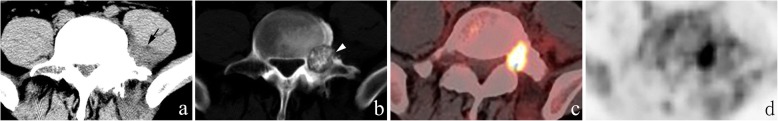
Osteoblastoma in a 28-year-old male, Enneking stage 2 on CT. **a** Axial CT soft tissue window shows a hypo-dense soft tissue mass (arrow) medial to the left psoas major muscle, which demonstrates the flare phenomenon. **b** Axial CT bone window shows expansile bone destruction in the lamina of L4 with a large quantity of matrix calcifications and sclerotic changes in the surrounding areas. Note the cortical breakthrough in the left pedicle (arrowhead). **c** and **d** F18-FDG PET/CT images show high uptake of FDG (SUVmax: 14.3) in the area of bone destruction, without FDG uptake abnormalities of the surrounding reactive sclerosis or inflamed tissues

**Fig. 4:**
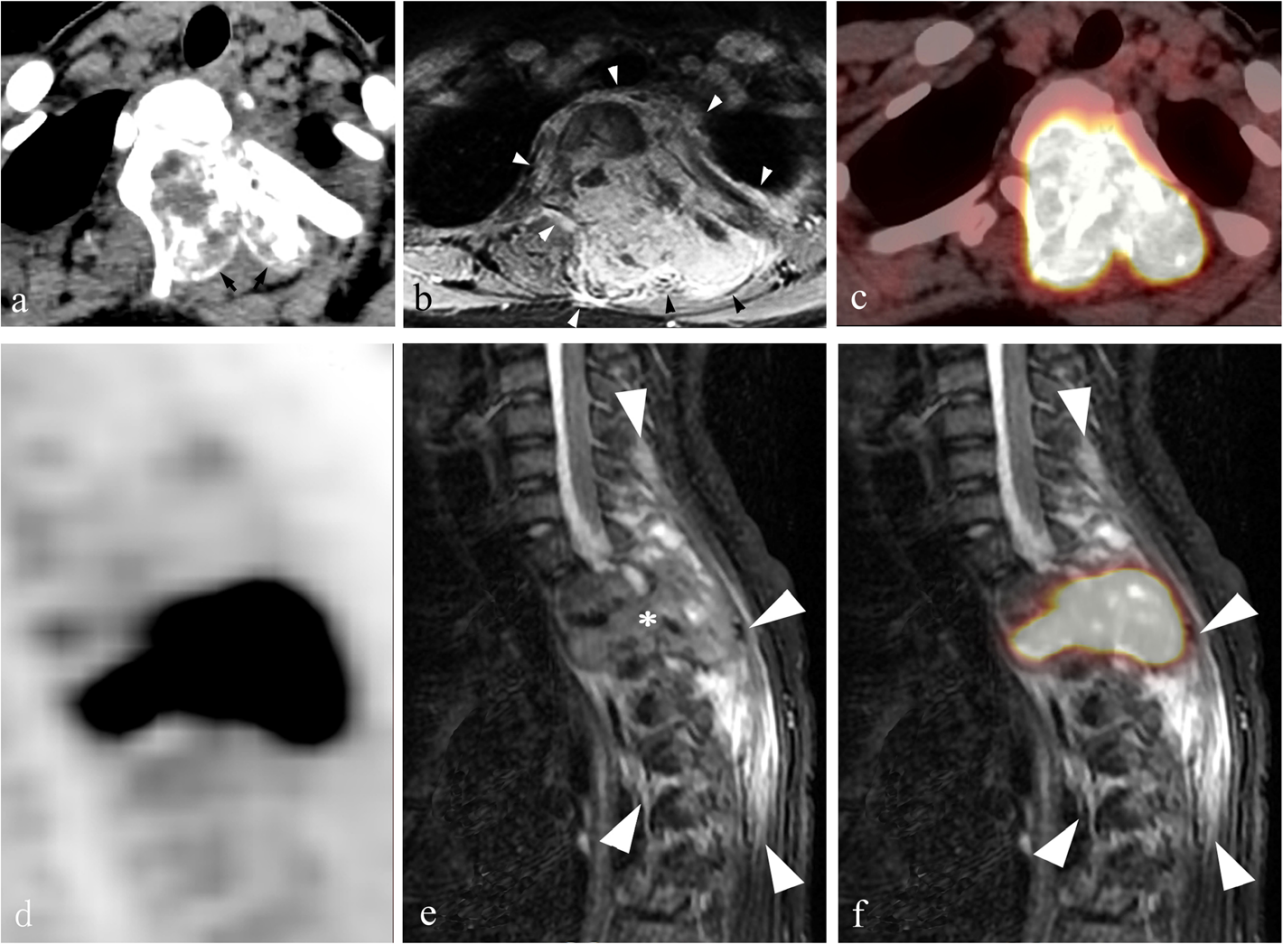
Aggressive osteoblastoma in a 16-year-old female, Enneking stage 3 on both CT and MRI. This case was diagnosed as Ewing’s sarcoma or osteosarcoma before needle biopsy. **a** Axial CT soft tissue window shows extensive osteolytic destruction in the vertebral body, left pedicle and lamina of T2 vertebrae, with scattered internal nodular calcifications and a sclerotic rim (arrows). Note that the structures in the spinal canal are not clearly depicted. **b** Axial MRI contrast-enhanced T1WI shows a diffuse mass (arrowheads) with avid enhancement greater than that observed on CT, involving the T2 vertebral body and anterior soft tissues, bilateral lamina, spinous process, left pedicle, head and neck of the left 2nd rib and their surroundings, as well as soft tissues inside the spinal canal encapsulating the cord. **c** and **d** Axial and sagittal F18-FDG PET/CT images show both the mass and flares with high uptake of FDG (SUVmax: 15.7), which indicates tumor extension into the surrounding soft tissues. Note that PET does not clearly show the spinal cord due to its low spatial resolution. **e** Sagittal T2WI shows the tumor (asterisk) and extensively swollen tissues surrounding it that display the flare phenomenon (arrowheads). **f** Co-registered PET and T2WI sagittal image shows that a lesion visible on MRI (arrowheads) is significantly larger than the foci of high uptake on PET-CT, which indicates the existence of inflammatory issues in the flares

- Size: A maximum diameter ranging from 22 to 68 mm and mean diameter of 38 mm.
- Location: Out of 21 total, 13 cases were mainly located in the posterior elements with extension to the vertebral body (Fig. 1), 6 cases involved only posterior elements (Fig. 2), one was limited to the spinous process, and one only involved the vertebral body.
- Shape: Lobulated (*n* = 7), irregular (*n* = 11), and oval (*n* = 3).
- Density/signal intensity: The solid parts of the tumor showed iso-density or hypo-density mass on non-contrast CT, hypo-intensity on T1WI, hyper-intensity on T2WI, and variable enhancement patterns. Spotted or nodular calcifications were found within all tumors. There was one case accompanied by an aneurysmal bone cyst (ABC).
- Sclerotic rim: Complete (*n* = 12), incomplete (*n* = 8) and absent (*n* = 1)..
- The “flare phenomenon”: Thin (< 5 mm) and thick (> 5 mm) flare tissues were noted in 11 cases and 6 cases on CT scans, respectively, and the other 4 cases did not present flares. When flares were examined via MRI, 18 of the 19 cases showed thick flares, and the other one showed thin flares. They appeared as diffuse signal abnormalities within adjacent vertebrae, surrounding paraspinous soft tissues and ribs within proximity, with hypo-density on non-contrast CT, hypo-intensity on T1WI, and hyper-intensity on T2WI (Figs. 1 and 2). All affected regions showed remarkable enhancement after administration of contrast agent.
- ^18^F-FDG PET: All tumors (*n* = 8) showed a nodular or lobulated area of avid uptake (Fig. 3) with an SUVmax of 12.3–16.0. Eight cases with the “flare phenomenon” showed low metabolism in the region of flare in 7 cases and coexistence of high and low metabolism (Fig. 4) in the other.

### Tumor staging

Based on CT, 3 of 21 cases were considered Enneking stage 1, 12 cases as stage 2, and 6 cases as stage 3. Based on MRI, 1 of 19 cases was considered Enneking stage 2 and the remaining 18 cases as stage 3. Consistency between CT and MRI was reached in 6 of 19 cases (31.6%), but the staging based on MRI was otherwise higher than that on CT in the other cases, as shown in Table 1.

## Discussion

### General radiological features of OB

a) Size: Size is an important differentiator between osteoblastomas (OB) and osteoid osteomas, with the former typically measuring greater than 2 cm in size [13]. All of the OB tumors in this study were larger than 2 cm. b) Density/signal intensity: Due to the large amount of loose fibrous connective tissue and abundant vascular matrix, most OBs demonstrate hypo- to iso-attenuation on non-contrast CT images, slight hypo- to iso-intensity on T1WI, and hyper-intensity on T2WI, with variable enhancement patterns on contrast-enhanced T1WI [14]. c) Calcification: Intratumoral calcification is a common sign of OB and was noted in all cases in our cohort. d) The sclerotic rim: As shown in the present study, 20/21 cases had marked sclerotic rims. Of note, completeness of the rim could not differentiate between conventional and aggressive OB. e) Location: Spinal OB mostly involves the posterior elements of the spine. In the report by Anne et al, 49/102 cases of OB were confined to the posterior elements [1]. However, extension from the posterior elements into the vertebral body might be more common [2, 15]. In the present study, the majority of OBs not only involved the posterior element but also the vertebral body. According to the literature and our present study, it is apparent that intratumoral calcification, presence of a sclerotic rim, and predominant involvement of the posterior elements are important features to consider in the diagnosis of spinal OB.

### The “flare phenomenon”

The flare phenomenon is one of the most interesting features of spinal OB, and is characterized by diffuse swelling of the marrow of adjacent vertebrae and ribs, as well as soft tissues in paravertebral regions and/or within the spinal canal [14]. Pathologically, the “flare phenomenon” reflects nonspecific inflammatory edema mixed with loose fibrous tissue, hypervascularity, mature lymphocytes, and plasma cells [10]. This inflammatory response is thought to be caused by tumor-derived prostaglandins [16]. The abnormal signals in the “flare area” can completely resolve after the tumor is surgically resected [11, 17]. In general, non-contrast CT is not a useful way to confidently detect flares. In contrast, MRI T2WI or fat-suppressed T2WI can clearly visualize them, and usually shows obvious enhancement following administration of contrast agents [2] (Figs. 1 and 2). Notably, flares can lead to an overestimation of the extent or Enneking staging of the tumor, especially on MRI. As exemplified in this study, the staging based on MRI was higher than on CT in ~ 70% of the cases examined. Furthermore, flares can result in the overestimation of the degree of malignancy of the tumor, leading to a misdiagnosis of osteosarcoma, Ewing’s sarcoma, or lymphoma [11, 18–20]. In fact, the current Enneking staging system involves only CT or MRI and is challenged as a clinical treatment guide for spinal OBs [21]. Chan et al. reported only a moderate level of agreement (Fleiss k coefficient = 0.47) between independent observers who recommended surgical resection of primary spinal tumors according to the Enneking staging system [21]. In addition, not all adjacent soft tissue masses in the setting of OB are afflicted by inflammation. Shaikh et al. [11] reported 2/12 cases of osteoblastomas where tumor tissue was found in the regions surrounding the diseased bone, as in Case 16 of the present study (Fig. 4). In short, owing to the flare phenomenon, it is difficult to accurately diagnose spinal OB based only on CT and/or MRI.

### The diagnostic value of PET

PET reflects the metabolic level of tissues and is very helpful for the qualitative diagnosis of tumors. Generally speaking, SUV_max_ > 2.5 mostly indicates a malignant tumor, SUVmax of 2.0–2.5 indicates a borderline lesion, and SUVmax < 2.0 is suggestive of a benign lesion. However, all reported cases of OB with ^18^F-FDG PET data uniformly showed hypermetabolic foci, with an average SUVmax of 7.0 [3, 4, 6–9]. In line with the literature, the average SUVmax of 8 cases in this present study was up to 14.6. As reported by Kusai et al., the metabolic rate of glucose does not necessarily correlate with biologic aggressiveness of bone tumors [6]. Generally, osteoblastoma has high osteogenic activity, which consequently leads to high uptake of ^18^F-FDG by tumors [14]. Thus, it might be concluded that PET cannot be used to distinguish between conventional and aggressive OB. With regard to the flare phenomenon, we could not find any published data on their detection using PET scans. In our study, among 8 cases with flares on CT and/or MRI, 7 showed no uptake of ^18^F-FDG in the region of “flare”, and the other showed coexistence of high and low metabolism (Fig. 4). This indicated that PET could be very helpful in the accurate characterization of a flare phenomenon, and thus can make up for the shortcomings of MRI.

### Multi-modality evaluation

**Fig. 5:**
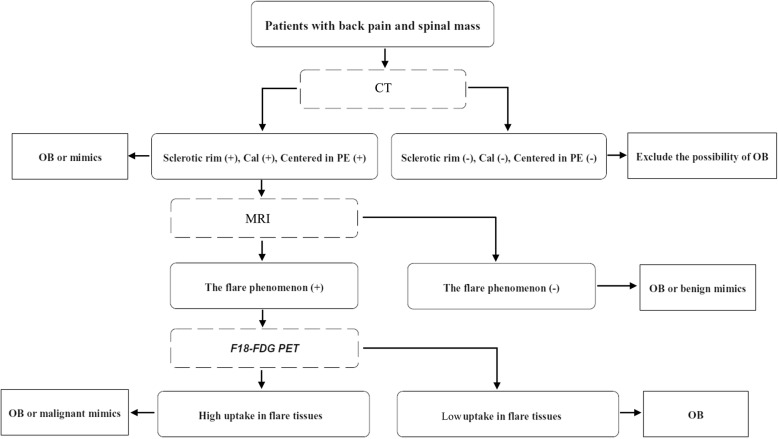
Proposed diagnostic work-up for patients suspected of having spinal osteoblastoma. OB, osteoblastoma; Cal, calcification; PE, posterior elements

It is clear that CT, MRI and PET have their own advantages and disadvantages in diagnosing and staging OB. Although CT can clearly show any sclerotic rim and calcifications of tumor matrix, it is subject to underestimation of the extent of the lesion. MRI can depict the flare phenomenon very clearly and help to provide a more complete vision of the lesions. At the same time, MRI is prone to overestimating the degree of malignancy and Enneking tumor stage, and thus the preoperative evaluation based on MRI alone may lead to unnecessary resection of bone [22] and other therapeutic interventions. ^18^F-FDG PET has the advantage of determining metabolic characteristics of the tumor and its true boundaries, but it is subject to overestimation of malignancy of the lesion and can fail to properly characterize inflammation in tissues surrounding the tumor. Therefore, combining the full potential of these three imaging techniques is critical to improving the accurate diagnosis and treatment options for patients with spinal OB. A proposed flow chart for patient care is provided in Fig. 5. For patients with suspected bone tumor of the spine, non-contrast CT would initially be recommended to identify any sclerotic rim, intratumoral calcifications and location of the lesion, followed by an MRI to look for the flare phenomenon, and then an ^18^F-FDG PET to determine the metabolic level of the flare tissues. When the flare tissues show low metabolism (uptake) on PET, the diagnosis of conventional spinal OB is strongly suggested, and the extent of surgical resection can be designed based on the sclerotic rim shown on CT. Meanwhile, when the clinical situation permits (e.g., no obvious spinal cord compression by inflammation in the surrounding tissues), the adjacent tissues in the spinal canal might be preserved. On the other hand, tissues surrounding the tumor that give a “flare signal” (or high metabolism) suggests OB with extraosseous extension or other malignant tumors, such as osteosarcoma or Ewing sarcoma, and the exact area(s) of surgical resection should be determined by MRI, where a more obvious flare signal can guide complete resection of the afflicted tissue.

### Limitations

While the sample size of the current study is relatively small, especially for PET, our report is the largest cohort of spinal OB with PET data reported in the literature so far. In addition, due to the retrospective nature of this study, the work-up we proposed had not been integrated into clinical practice. Not all patients underwent total en bloc resection, which may affect the accuracy of the results. Therefore, a large-scale, multi-center and prospective study is needed to confirm the efficacy and applicability of our current findings.

## Conclusions

In conclusion, spinal osteoblastoma is a rare tumor with several distinct radiologic features. However, it is difficult to obtain an accurate quantitative and qualitative diagnosis based on CT, MRI or PET alone, which is mainly due to the influence of the flare phenomenon. For patients suspected to have spinal osteoblastoma, using CT, MRI and PET in combination could help to optimize preoperative diagnosis, and determine the most suitable options for treatment and management of these tumors.

## Data Availability

The datasets used and analyzed during the current study are available from the corresponding author on reasonable request.

## Abbreviations

OB: Osteoblastoma
PACS: Picture archiving and communication system
SUVmax: Maximum standard uptake value
ABC: Aneurysmal bone cyst

## References

[1] Versteeg AL, Dea N, Boriani S, Varga PP, Luzzati A, Fehlings MG, Blisky MH, Rhines LD, Reynolds JJ, Dekutoski MB, Gokaslan ZL, Germscheid NM (2017). Fisher CG surgical management of spinal osteoblastomas. J Neurosurgical Spine.

[2] Galgano MA, Goulart CR, Iwenofu H, Chin LS, Lavelle W, Mendel E (2016). Osteoblastomas of the spine: a comprehensive review. Neurosurgical Focus.

[3] Reynolds JJ, Rothenfluh DA, Athanasou N, Wilson S, Kieser DC (2017). Neoadjuvant denosumab for the treatment of a sacral osteoblastoma. Eur Spine J.

[4] Shimizu N, Sakata K, Yamamoto I (2006). Benign osteoblastoma of the temporal bone: case report and review of the literature. Surg Neurol.

[5] Tang H, Zou D, Chen W (2011). Imaging diagnosis of aggressive osteoblastoma. J Clin Radiol.

[6] Al-Muqbel KM, Al-Omari MH, Audat ZA, Alqudah MA (2013). Osteoblastoma is a metabolically active benign bone tumor on 18F-FDG PET imaging. J Nuclear Med Technol.

[7] Jeong YJ, Sohn MH, Lim ST, Kim DW, Jeong HJ, Jang KY, Yim CY (2011). F-18 FDG PET/CT and Tc-88m MDP 3-phase bone scan findings with pathologic correlation. Clin Nucl Med.

[8] Imeperiale A, Moser T, Ben-Sellem D, Mertz L, Gangi A, Constantinesco A (2009). Osteoblastoma and osteoid osteoma: Morphofunctional characterization by MRI and dynamic F-18 FDG PET/CT before and after radiofrequency ablation. Clin Nucl Med.

[9] Strobel K, Merwald M, Huellner M, Zenklusen HR, Kuttenberger J (2013). Osteoblastoma of the mandible mimicking osteosarcoma in FDG PET/CT imaging. Clin Nucl Med.

[10] Shaikh MI, Saifuddin A, Pringle J, Natali C, Sherazi Z (1999). Spinal osteoblastoma: CT and MR imaging with pathological correlation. Skelet Radiol.

[11] Enneking WF (1986). A system of staging musculoskeletal neoplasms. Clin Orthop Relat Res.

[12] Lucas DR, Unni KK, Mcleod RA, O’Connor MI, Sim FH (1994). Osteoblastoma: clinicopathologic study of 306 cases. Hum Pathol.

[13] Abdel Razek AA, Csstillo M (2010). Imaging appearance of primary bony tumors and pseudo-tumors of the spine. J Neuroradiol.

[14] Loizaga JM, Calvo M, Lopez BF, Martinez Tello FJ, Perez VJ (1993). Osteoblastoma and osteoid osteoma: clinical and morphological features of 162 cases. Pathol Res Pract.

[15] Crim JR, Mirra JM, Eckard JJ, Seeger LL (1990). Widespread inflammatory reponse to osteoblastoma: the flare phenomenon. Radiology..

[16] Yamamura S, Sato K, Sugiura H, Katagiri H, Fukatsu H, Iwata H (1997). Prostaglandin levels of primary bone tumor tissues correlate with peritumoral edema demonstrated by magnetic resonance imaging. Cancer..

[17] Chakrapani SD, Grim K, Kaimaktchiev V, Anderson JC (2008). Osteoblastoma of the spine with discordant magnetic resonance imaging and computed tomography imaging features in a child. Spine..

[18] Kroon HM, Bloem JL, Holscher HC, van der Woude HJ, Reijnierse M, Taminaiau AH (1994). MR imaging of edema accompanying benign and malignant bone tumors. Skelet Radiol.

[19] Orguc S, Arkun R (2014). Primary tumors of the spine. Semin Musculoskelet Radiol.

[20] Wan Y, Zhao W, Jiang Y, Liu D, Meng G, Cai Y (2014). β-catenin is a valuable marker for differential diagnosis of osteoblastoma and osteosarcoma. Hum Pathol.

[21] Chan P, Boriani S, Fourney DR, Biagini R, Dekutoski MB, Fehlings MG, Ryken TC, Gokaslan ZL, Vrionis FD, Harrop JS, Schmidt MH, Vialle LR, Gerszten PC, Ondra SL, Pratt SR, Fisher CG (2009). An assessment of the reliability of the Enneking and Weinstein-Boriani-Biagini classifications for staging of primary spinal tumors by the Spine Oncology Study Group. Spine (Phila Pa 1976).

[22] Patel AJ, Fox BD, Fahim DK, Fulkerson DH, Whitehead WE, Curry DJ, Luerssen TG, Jea A (2011). A clinlcopathologic correlation in osteoblastoma of the spine in a child. J Clin Neurosci.

